# Is health coaching effective in changing the health status and behaviour of prisoners?—a systematic review protocol

**DOI:** 10.1186/s13643-017-0524-5

**Published:** 2017-07-03

**Authors:** Nadja Almondes, Denise Downie, Ayse B. Cinar, Derek Richards, Ruth Freeman

**Affiliations:** 1 Dental Health Services Research Unit, Dundee Dental Hospital and School, University of Dundee, 9th Floor, Park Place, Dundee, DD1 4HN UK; 2 Scottish Prison Service, SPS College, Brightons, Falkirk, FK2 0AD UK; 3 Public Health Department, NHS Forth Valley and Tayside, Carseview House, Castle Business Park, Stirling, FK9 4SW UK

**Keywords:** Health coaching, Wellness coaching, Health behaviour, Prison, Prisoners

## Abstract

**Background:**

This is a protocol for a systematic review of the impact of health coaching on changing the health behaviour of offenders. Prisoners are more likely to suffer from health-related issues when compared to the general population. Health coaching has been shown to influence health outcomes of patients with chronic conditions. This review, therefore, aims to assess the effectiveness of health coaching interventions on the health of adolescent and adult offenders in custodial institutions.

**Methods:**

We plan to conduct a systematic review of the current literature on health coaching interventions delivered in the prison setting. We will include randomised controlled trials and observational studies that compare health coaching to the usual care or other alternative interventions. The ideal interventions will be delivered either by health professionals or peer coaches, and the outcomes extracted in the data collection will be disease-specific, clients’ life and self-management skills, behavioural and psychosocial outcomes. If appropriate, a meta-analysis of the data collected will be carried out on the last stage of the review.

**Discussion:**

This systematic review will identify and gather evidence on the impact of health coaching interventions delivered in the prison setting and can function as a supporting material for health professionals, prison staff, the healthcare system, and public health departments when considering delivering health coaching.

**Systematic review registration:**

PROSPERO CRD42016053237.

**Electronic supplementary material:**

The online version of this article (doi:10.1186/s13643-017-0524-5) contains supplementary material, which is available to authorized users.

## Background

Prisoners suffer from a variety of health-related issues, which tend to be of higher prevalence when compared to the general population [1]. As the majority of offenders come from the most underprivileged sections of society, lower educational levels, high-risk health behaviours and less frequent access to health care contribute to their higher prevalence of diseases [2, 3]. On the same note, lifestyle-related diseases, for example non-communicable conditions such as cardiovascular diseases, cancer, chronic respiratory diseases and diabetes [4], that impact upon the well-being of inmates are the most prevalent and easily preventable conditions that are affected by behaviour change [5]. There exists a need, therefore, to address the prisoners’ health behaviours to manage and prevent such chronic disease states.

The *Health in prisons—A WHO guide to the essentials in prison health* calls for prison health programmes to address health promotion, rather than simply offering health care [6]. The guide also points out that the prison environment offers access to a group of people that would otherwise be harder to reach by health professionals. Health coaching (HC) could be used as a tool to deliver health knowledge and reduce the prisoner’s high-risk health behaviours.

HC has been defined in many different ways over the years. Wolever et al. [7] conducted a systematic review to find a consensus on the definition of health coaching. According to their data, HC may be defined broadly as a patient-centred approach to promoting behaviour change. HC should focus on working towards goals, and those goals should be at least partially determined by the client. HC interventions should also use content education and self-monitoring to increase accountability, as well as including an interpersonal relationship with a coach, as conceptualised here as a logic model (see Table 1).

**Table 1:** Health coaching logic model

Situation	Input	Outputs		Outcomes
		Activities	Participation	
Closed states Open prisons	Health professionals Trained coaches Peer coaches	Goal setting Content education Active learning process Behavioural self-monitoring Interpersonal relationship with a coach	Adult prisoners Young offenders	Disease-specific (such as changes in blood pressure, blood sugar control, cholesterol, cardiovascular risk factor, control of pain, weight loss or changes in body mass index (BMI) and waist circumference) Life and self-management skills (self-efficacy and ability to perform daily activities) Behavioural and psychosocial outcomes (motivation to self-management, self-confidence, recovery expectation, medical adherence, lifestyle choices and self-reported emotional well-being)

The current evidence for the effectiveness of health coaching interventions varies. A systematic review recently published showed that HC significantly improved physiological, behavioural, psychological and social outcomes of patients with chronic conditions [8]. When reviewing the effect of HC on low back pain management, no significant between-groups improvement was found for physical outcomes [9]; however, patients on the HC group appeared to have a more positive attitude and were more compliant with treatments.

The aim of this systematic review is to explore the impact of HC interventions for prisoners regarding health outcomes by applying a broad definition of health coaching and, whenever possible, comparing to standard clinical care or other alternative interventions. The health outcomes would include behavioural, psychologic, disease-specific and physiologic, as well as any other measures of patients’ capacity that can be presented as having an impact on the patient’s health condition. Ideally, the characteristics of the most effective coaching interventions will be identified, regarding 1. duration and frequency of the sessions, 2. delivery format and 3. coach qualification.

To this end, this proposed systematic review will answer the following questions:

1. Is HC effective in changing the health status of prisoners, when compared to standard care?
2. What are the desirable characteristics for designing successful HC interventions, regarding their duration, the frequency of coaching sessions, delivery format and coach qualification.

## Methods

### Study design

A systematic review and, if possible, a meta-analysis will be conducted following the Preferred Reporting Items for Systematic Reviews and Meta-Analyses (PRISMA) statement [10].

### Study eligibility

#### Type of studies

Randomised controlled trials, controlled before-after studies, prospective and retrospective comparative cohort studies, case-control studies, cross-sectional studies, case series and case reports, published in English, Portuguese or Spanish, will be included in this review. Moreover, to broaden the scope of this review, non-randomised studies will also be considered, providing that they fit with the coaching definition stated in Wolever et al. [7].

#### Types of participants

Residents of prisons, correctional facilities or young offenders’ institutions, aged 16 years old or older. These institutions can include closed and open prison estates.

#### Types of interventions

Studies that implement a coaching intervention in the prison setting will be included. The coaching definition should fit the characteristics presented by Wolever et al. [7], which are patient-centred intervention, with the goals at least partially defined by the participant; active learning process and content education focusing on the goal previously set; stimulation of behavioural self-monitoring; and an interpersonal relationship with a coach. Interventions delivered by either health professionals, trained coaches or peer coaches will be considered, peer-based interventions being defined as activities where prisoners offer advice, education and support to other prisoners [11].

#### Types of comparators

Studies that compare HC interventions with standard clinical care or alternative interventions will be included in this review.

#### Types of outcome measures

Three types of health outcomes will be extracted: disease-specific outcomes, such as, but not limited to, changes in blood pressure, blood sugar control (for diabetic patients), cholesterol, cardiovascular risk factor, control of pain, weight loss or changes in body mass index (BMI) and waist circumference; patients’ life and self-management skills including self-efficacy and ability to perform daily activities; behavioural and psychosocial outcomes including motivation to self-management, self-confidence, recovery expectation, medical adherence, lifestyle choices (such as physical exercises, eating habits and smoking) and self-reported emotional well-being. All outcomes previously listed will be considered main outcomes, as goal setting for HC varies largely on the clients’ self-perception. No secondary outcomes will be extracted for this review.

### Search strategy

The search will be conducted in relevant electronic databases and also by visually scanning reference lists from relevant studies, hand searching key journals and conference proceedings, and by contacting study authors and experts on the subject. The electronic databases to be searched are PubMed/MEDLINE (NCBI interface), CINAHL, PsycINFO, ASSIA, Cochrane Central Register of Controlled Trials (Wiley interface), Web of Science, Scopus and LILACS. The search will be developed using medical subject headings (MeSH) and text words related to counselling, prisoners, health coaching, health promotion and health education. A draft MEDLINE search strategy is included in an additional file (see Additional file 1). After the MEDLINE strategy is finalised, it will be adapted to the other databases listed. When the electronic database search is finished, the references of selected studies will also be searched. When the full search strategy is completed, a topic expert will be asked to check the list of selected publications to identify any known missing studies. Searched results will be managed using the software EndNote X7.

### Selection of studies

Duplicates will be removed using EndNote’s duplicate identification tool, and then manually as required. The remaining studies will then be screened in two stages. The first stage will consist of a title and abstract screening conducted by a solo reviewer; any studies that are not in a prison setting and do not include a health outcome will be excluded at this phase. The second stage, a full-text screening, will be conducted independently and in duplicate by two peer reviewers, which will follow an exclusion proforma, included in an additional file (see Additional file 2). Any discrepancies in the selection process will first be discussed between the two peer reviewers, and, if no consensus is achieved, a third reviewer will be called to arbitrate. The results of the search and the process of screening will be presented in a study flow diagram, following the PRISMA template [10]. Included studies will be presented in a “Characteristics of included studies” table, containing their methods, participants, interventions, outcomes and notes.

### Data collection and management

A standardised data collection form (see Additional file 3) will be used to extract data from the included studies for assessment of study quality and evidence synthesis. Extracted information will include population characteristics (number of participants, age range, gender, nationalities and baseline characteristics), study setting, details of the intervention (control conditions, number of sessions delivered, duration and frequency of sessions, delivery format and coach qualifications), methodology (study design and duration, characteristics of the recruitment and completion rates) and outcomes (types of outcomes measure and measurement times). The collection of data will be performed by two reviewers independently and in duplicate and discrepancies will be resolved by a third. Missing data will be requested from study authors. The data extracted will be managed using a specially developed proforma.

### Risk of bias assessment

The RoB (Risk of Bias) 2.0 tool [12], a revised tool to assess the risk of bias in randomised trials, will be used as appropriate, and the adequate form will be used depending on the trial format (individually randomised parallel group trials, cluster randomised parallel group trials or individually randomised cross-over trials). To assess the risk of bias of non-randomised studies, The ROBINS-I tool (Risk Of Bias In Non-randomized Studies—of Interventions) [13] will be applied.

To analyse the presence of publication bias, protocols for the randomised controlled trials will be checked for the date of publication to establish if this preceded the publication of results from studies; we will further determine if the outcomes were selectively reported and if the sample size was adequate. In the event more than 10 studies are available for reviewing, a funnel plot will be used to explore the potential of publication bias, with the relevant outcomes being selected inductively according to this study’s findings.

### Data synthesis

The data will be synthesised and presented in a narrative form. A descriptive paragraph will be provided with the results of each study. Whenever possible, the studies will be grouped into clusters, according to their similarities, regarding the characteristics of the intervention or population group, for example. The results will then be gathered into a “Summary of findings” table, which will contain any important outcomes, a measure of the burden of these outcomes, the magnitude of the effect, the number of participants and studies addressing each outcome, a grade for the quality of evidence for each outcome and comments. An Intervention Component Analysis (ICA) [14] will be used to identify the desirable characteristics for design successful HC interventions. The ICA will be performed in two stages: the first stage will seek to identify the differences between interventions, which will be done through a narrative analysis of the intervention’s characteristics, and the second stage will establish which characteristics of the intervention appear to explain the differences in outcomes, by mapping the features and emergent themes from the studies data.

### Statistical analysis

If appropriate, a meta-analysis will be conducted using RevMan 5.35 [15] with the random-effects model as a framework, which is the most suitable method to incorporate studies with significant heterogeneity [16], as we anticipate this will be the case with regard to characteristics of the population and the intervention. The between-study variability will be evaluated using the *I* ^2^ index [17]. The outcomes for the meta-analyses will be those previously selected for the publication bias assessment. Meta-analysis will only be considered when two or more studies report the same outcome measure.

Subgroup analysis will be used to explore the sources of heterogeneity, based on participants demographic characteristics (age, gender, nationality, baseline characteristics), characteristics of the intervention (number, duration and frequency of sessions, delivery format and coach qualifications) and follow-up period.

### Quality of evidence

Quantitative data will be evaluated using the Grading of Recommendations Assessment, Development and Evaluation (GRADE) framework for quality of evidence [18].

## Discussion

Based on the evidence that shows the efficiency of health coaching in improving the health outcomes [8], it seems important to access the likelihood of this intervention being effective in a prison setting and in addressing a wider range of health concerns.

Overall, this report will act as supporting material for health professionals, prison staff, the healthcare system and public health departments when considering whether to deliver health coaching interventions in the prison setting. With more evidence on the effectiveness of this approach, it could become an important guide for developing behavioural interventions, in particular, coaching, to address the general health issues and to promote well-being among prisoners.

This protocol was developed in accordance with the PRISMA-P statement (see Additional file 4).

## Additional files

Additional file 1: Draft of MEDLINE search shows the draft for search strategy of MEDLINE using NCBI interface. (DOCX 11 kb)

Additional file 2: Article exclusion questions show the proforma to be used for articles exclusion. (DOCX 13 kb)

Additional file 3: Article inclusion form shows the form to be used to extract data from articles included in the study. (DOCX 12 kb)

Additional file 4: PRISMA-P checklist acknowledges that we have met key requirements for Systematic review protocols. (DOCX 29 kb)

Additional file 5: Evidence of funding, award number: 290.804485. (PDF 46 kb)

## Abbreviations

BMI: Body mass index
CASP: Critical appraisal skills programme
GRADE: Grading of Recommendations Assessment, Development and Evaluation
HC: Health coaching
ICA: Intervention Component Analysis
MeSH: Medical subject headings
PRISMA: Preferred Reporting Items for Systematic Reviews and Meta-Analyses
PROSPERO: International Prospective Register of Systematic Reviews
RoB: Risk of bias
ROBINS-I tool: Risk Of Bias In Non-randomized Studies—of Interventions
