# Supplementary material for: Is health coaching effective in changing the health status and behaviour of prisoners?—a systematic review protocol
Source: Syst Rev. 2017 Jul 3;6:127. doi: 10.1186/s13643-017-0524-5 (PMC5496214; doi:10.1186/s13643-017-0524-5)
Supplement: Supplementary file 1 — Draft of MEDLINE search shows the draft for search strategy of MEDLINE using NCBI interface. (DOCX 11 kb) [file 13643_2017_524_MOESM1_ESM.docx]

Draft MEDLINE search – NCBI interface

S1. MH health promotion

S2. MH health education

S3. MH health behavior

S4. MH life style

S5. MH quality of life

S6. MH attitude to health

S7. MH health knowledge, attitudes, practice

S8. MH self care

S9. MH holistic care

S10. MH exercise therapy

S11. S1 OR S2 OR S3 OR S4 OR S5 OR S6 OR S7 OR S8 OR S9 OR S10

S12. wellness program*

S13. Coach*

S14. health coach*

S15. Life style coach*

S16. Lifestyle coach*

S17. Wellness coach*

S18. S12 OR S13 OR S14 OR S15 OR S16 OR S17

S19. Prison*

S20. Inmate*

S21. Offend*

S22. Criminal justice

S24. Correctional facilities

S25. S19 OR S20 OR S21 OR S22 OR S23 OR S24

S26. S11 AND S18 AND S25
