# Supplementary material for: Is health coaching effective in changing the health status and behaviour of prisoners?—a systematic review protocol
Source: Syst Rev. 2017 Jul 3;6:127. doi: 10.1186/s13643-017-0524-5 (PMC5496214; doi:10.1186/s13643-017-0524-5)
Supplement: Supplementary file 2 — Article exclusion questions show the proforma to be used for articles exclusion. (DOCX 13 kb) [file 13643_2017_524_MOESM2_ESM.docx]

Article Exclusion Questions

| Title of Project |  |
| --- | --- |

| Paper Number |  |
| --- | --- |
| First Author |  |
| Title |  |
| Year Published |  |

Any red boxes means paper is discounted

|  | Yes | No |
| --- | --- | --- |
| 1. Language other than English, Portuguese or Spanish? |  |  |
| 1. Article other than original data? |  |  |
| 1. The patient did not set his own goals? |  |  |
| 1. The intervention did not include active-learning process? |  |  |
| 1. The intervention did not stimulate behavioural self-monitoring? |  |  |
| 1. Did intervention not include an interpersonal relationship with a coach? |  |  |
| Paper excluded? |  |  |
