# Supplementary material for: Is health coaching effective in changing the health status and behaviour of prisoners?—a systematic review protocol
Source: Syst Rev. 2017 Jul 3;6:127. doi: 10.1186/s13643-017-0524-5 (PMC5496214; doi:10.1186/s13643-017-0524-5)
Supplement: Supplementary file 3 — Article inclusion form shows the form to be used to extract data from articles included in the study. (DOCX 12 kb) [file 13643_2017_524_MOESM3_ESM.docx]

Article Inclusion Form

| Paper Number |  |
| --- | --- |
| First Author |  |
| Title |  |
| Year Published |  |

Study Population

| Number of participants |  |
| --- | --- |
| Age |  |
| Gender |  |
| Nationalities |  |
| Baseline characteristics |  |

Study Setting

| Setting |  |
| --- | --- |

Intervention

| Control conditions |  |
| --- | --- |
| Number of sessions |  |
| Duration of sessions |  |
| Frequency of sessions |  |
| Delivery format |  |
| Coach qualifications |  |

Methods

| Design of study |  |
| --- | --- |
| Duration |  |
| Recruitment |  |
| Completion rates |  |

Outcomes

| Types of outcomes measured |  |
| --- | --- |
| Times of measurements |  |
