# Supplementary material for: Is health coaching effective in changing the health status and behaviour of prisoners?—a systematic review protocol
Source: Syst Rev. 2017 Jul 3;6:127. doi: 10.1186/s13643-017-0524-5 (PMC5496214; doi:10.1186/s13643-017-0524-5)
Supplement: Supplementary file 5 — Evidence of funding, award number: 290.804485. (PDF 46 kb) [file 13643_2017_524_MOESM5_ESM.pdf]

UNIVERSITY OF DUNDEE

Finance Office

10924

Invoice/Claim No. 290.804485/1512
RNHSF94SW

18 July 2016

Derek Richards
Consultant in Dental Public Health
Forth Valley NHS
Public Health Department
Carseview House
Castle Business Park
Stirling
FK9 4SW

Researcher Professor R Freeman
Your Ref Prison Study
Title Scottish Oral Health Improvement in Prison Group (SOHIPP)
Customer Code: RNHSF94SW

April 2016 – March 2017

National Oral Health Improvement in Prisons Project Recurrent Funding	**37,763**
**Total Amount Claimed**	**£ 37,763**

Certified By : C.H. Strachan **Research Accountant**

Remittances quoting **290.804485/1512** should be made payable to **"The University of Dundee"** and addressed to **The Research Finance Office.**

Enquiries should be made to:
Miss Cheryl Chan
Telephone: (01382) 388139 (Direct Line)
e-mail: s.x.chan@dundee.ac.uk

VAT Registration No: 270 9397 31

Bank Details:
Royal Bank of Scotland
3 High Street
Dundee DD1 9LY
Sort Code: 83-50-00
Account Code: 00279732
Swift Code: RBOSGB2L
IBAN: GB13RBOS83500000279732
